# *N*-[(2*S*)-4-Chloro-2-(l-menthyloxy)-5-oxo-2,5-dihydro-3-furyl]-l-alanine

**DOI:** 10.1107/S1600536809012720

**Published:** 2009-04-10

**Authors:** Zhao-yang Li, Xiu-mei Song, Zhao-yang Wang, Kai Yang

**Affiliations:** a School of Chemistry and Environment, South China Normal University, Guangzhou 510006, People’s Republic of China

## Abstract

The title compound, C_17_H_26_ClNO_5_, was prepared *via* a tandem asymmetric Michael addition–elimination reaction of (5*S*)-3,4-dichloro-5-(l-menth­yloxy)furan-2(5*H*)-­one and l-alanine in the presence of potassium hydroxide. The five-membered furan­one ring is approximately planar while the six-membered menth­yloxy ring adopts a chair conformation. The crystal packing is stabilized by inter­molecular O—H⋯O and N—H⋯O hydrogen bonds.

## Related literature

For chiral 5-(l-menth­yloxy)furan-2(5*H*)-­ones as key building blocks in the synthesis of supra­molecules and important natural products, see: Feringa & De Jong (1988 ▶); He *et al.* (2006 ▶); Lattmann *et al.* (1999 ▶). For the use of 4-aminofuran-2(5*H*)-one in chemical, pharmaceutical and agrochemical research, see: Kimura *et al.* (2000 ▶); Tanoury *et al.* (2008 ▶). For a related structure, see: Wang *et al.* (2008 ▶). For the synthesis of the chiral synthon (5*S*)-3,4-dichloro-5-(l-menth­yloxy)furan-2(5*H*)-one, see: Chen & Geng (1993 ▶).
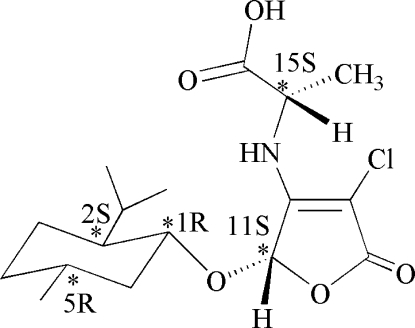


## Experimental

### 

#### Crystal data

C_17_H_26_ClNO_5_
*M* *_r_* = 359.84
Orthorhombic, 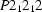

*a* = 11.2481 (15) Å
*b* = 19.642 (2) Å
*c* = 9.0668 (11) Å
*V* = 2003.1 (4) Å^3^
*Z* = 4
Mo *K*α radiation
μ = 0.21 mm^−1^
*T* = 293 K
0.30 × 0.23 × 0.18 mm

#### Data collection

Bruker APEXII area-detector diffractometer
Absorption correction: multi-scan (*SADABS*; Bruker, 2004 ▶) *T*_min_ = 0.761, *T*_max_ = 0.846 (expected range = 0.866–0.962)
10244 measured reflections
3534 independent reflections
2879 reflections with *I* > 2σ(*I*)
*R*_int_ = 0.037

#### Refinement

*R*[*F* ^2^ > 2σ(*F* ^2^)] = 0.046
*wR*(*F* ^2^) = 0.120
*S* = 1.08
3534 reflections
222 parameters
312 restraints
H-atom parameters constrained
Δρ_max_ = 0.33 e Å^−3^
Δρ_min_ = −0.34 e Å^−3^
Absolute structure: Flack (1983 ▶), 1499 Friedel pairs
Flack parameter: 0.00 (10)

### 

Data collection: *APEX2* (Bruker, 2004 ▶); cell refinement: *SAINT* (Bruker, 2004 ▶); data reduction: *SAINT*; program(s) used to solve structure: *SHELXS97* (Sheldrick, 2008 ▶); program(s) used to refine structure: *SHELXL97* (Sheldrick, 2008 ▶); molecular graphics: *XP* in *SHELXTL* (Sheldrick, 2008 ▶); software used to prepare material for publication: *SHELXL97*.

## Supplementary Material

Crystal structure: contains datablocks I, global. DOI: 10.1107/S1600536809012720/bq2134sup1.cif

Structure factors: contains datablocks I. DOI: 10.1107/S1600536809012720/bq2134Isup2.hkl

Additional supplementary materials:  crystallographic information; 3D view; checkCIF report

## Figures and Tables

**Table 1:** Hydrogen-bond geometry (Å, °)

*D*—H⋯*A*	*D*—H	H⋯*A*	*D*⋯*A*	*D*—H⋯*A*
N1—H1*A*⋯O5^i^	0.86	2.20	2.975 (4)	150
O4—H4⋯O3^ii^	0.82	1.86	2.655 (3)	164

Symmetry codes: (i) 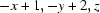; (ii) 
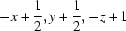
.

**Fig. 1.**
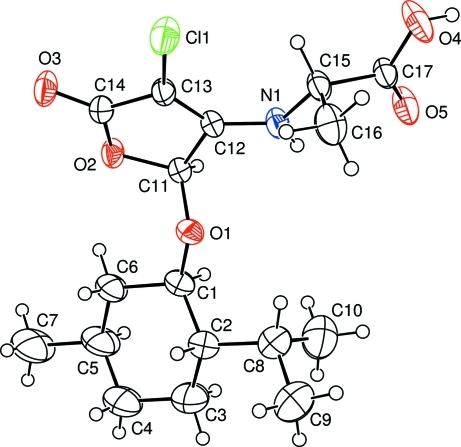
Displacement ellipsoid plot (40% probability level) of the title compound (I), with atom numbering of structurally unique non-H atoms and the H atoms.

**Fig. 2.**
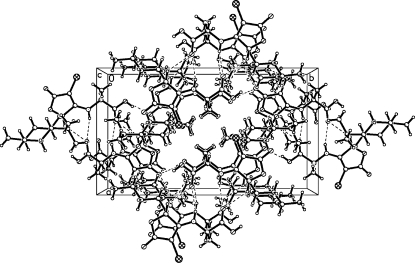
The packing diagram of the title compound (I) (viewed down the c axis).

## supplementary crystallographic information

### Comment

Chiral 5-(l-menthyloxy)-2(5*H*)-furanones have been extensively utilized as key building blocks in the synthesis of supramolecules and important natural products since 1980's (Feringa *et al.*, 1988; Lattmann *et al.*, 1999), especially in asymmetric synthesis (He *et al.*, 2006). At the same time, 4-amino-2(5*H*)-furanone is an attractive moiety in chemical, pharmaceutical and agrochemical research (Kimura *et al.*, 2000; Tanoury *et al.*, 2008). Herein we report the crystal structure of the title compound *N*-[3-chloro-5-(*S*)-(l-menthyloxy)-2(5*H*)-4-furanon-yl]-*L*-alanine, namely C_17_H_26_ClNO_5_, obtained *via* tandem asymmetric Michael addition-elimination reaction, with chiral building blocks 3,4-dichloro-5-(*S*)-(l-menthyloxy)-2(5*H*)-furanone and *L*-alanine.

The structure of the title compound (I) is illustrated in Fig. 1. The asymmetric unit of the title compound contains two independent rings: a five-membered furanone ring and a six-membered menthyloxy ring connected each other *via* C1—O1—C11 ether bond, having five chiral centers (C1(*R*), C2(S), C5(*R*), C11(S), C15(S)). The furanone ring of O2—C14—C13—C12—C11 is approximately planar, whereas the cyclohexane ring displays a chair conformation with three substituents occupying equatorial positions. In addition, the molecules of (I) are linked by O4—H4···O3 and N1—H1A···O5 intermolecular hydrogen bonds, giving rise to three-dimensional structure (Tab. 1 and Fig. 2). The bond distances and angles are mostly in agreement with the expected values (Wang *et al.*, 2008).

### Experimental

The chiral synthon 3,4-dichloro-5-(*S*)-(l-menthyloxy)-2(5*H*)-furanone was prepared according to the literature procedure (Chen *et al.*, 1993). After the mixture of *L*-alanine (0.18 g, 2.03 mmol) and potassium hydroxide (0.1274 g, 2.28 mmol) was dissolved in absolute ethyl alcohol under a nitrogen atmosphere, dichloromethane solution of 3,4-dichloro-5-(*S*)-(l-menthyloxy)-2(5*H*)-furanone (0.7494 g, 2.50 mmol) was added. The reaction was carried out under the stirring at room temperature for 24 h. Once the reaction was complete, the solvents were removed under reduced pressure. The residual solid was dissolved in dichloromethane, and the pH of the solution was adjusted to 3–4 with 15% of HCl aqueous solution. Then, the combined organic layers from extraction were concentrated under reduced pressure, and the crude product was purified by silica gel column chromatography with the gradient mixture of petroleum ether and ethyl acetate to give the product, yielding (I) 0.4348 g (59.5%). ^1^H NMR δ (400 MHz, CDCl_3_, TMS): 0.825–0.850 (3*H*, m, CH_3_-7), 0.901–0.955 (7*H*, m, CH-8, CH_3_-9, 10), 0.994–1.154 (2*H*, m, CH_2_-4), 1.340–1.439 (2*H*,m, CH-5, CH-2), 1.581–1.607 (3*H*, m, CH_3_-16), 1.630–1.700 (2*H*, m, CH_2_-3), 2.065–2.233 (2*H*, m, CH_2_-6), 3.520–3.602 (1*H*, m, CH-1), 4.771–4.864 (1*H*, m, CH-15), 5.195 (1*H*, s, NH), 5.704 (1*H*, s, CH-11), 8.950 (1*H*, s, COO-H); [α]^20°^_D_ = 53.96° (c 0.467, CH_3_CH_2_OH); ESI-MS, m/*z* (%): Calcd for C_17_H_27_ClNO_5_^+^ ([*M*+H]^+^): 360.16, Found: 360.32 (95.0).

### Refinement

All H atoms were positioned in calculated positions (O—H = 0.82Å; N—H = 0.86Å; C—H =0.96Å or 0.97Å or 0.98Å) and were refined using a riding model, with *U*_iso_(H) = 1.2 *U*_eq_(C) for methylene H atoms and *U*_iso_(H) = 1.5 *U*_eq_(O) for methyl or hydroxyl H atoms *U*_iso_(H) = 1.2 *U*_eq_(N) for amino H atoms.

### Crystal data

**Table:**

C_17_H_26_ClNO_5_	*F*(000) = 768.0
*M**_r_* = 359.84	*D*_x_ = 1.193 Mg m^−^^3^
Orthorhombic, *P*2_1_2_1_2	Mo *K*α radiation, λ = 0.71073 Å
Hall symbol: P 2 2ab	Cell parameters from 2460 reflections
*a* = 11.2481 (15) Å	θ = 2.3–20.5°
*b* = 19.642 (2) Å	µ = 0.21 mm^−^^1^
*c* = 9.0668 (11) Å	*T* = 293 K
*V* = 2003.1 (4) Å^3^	Block, colorless
*Z* = 4	0.30 × 0.23 × 0.18 mm

### Data collection

**Table:**

Bruker APEXII area-detector diffractometer	3534 independent reflections
Radiation source: fine-focus sealed tube	2879 reflections with *I* > 2σ(*I*)
graphite	*R*_int_ = 0.037
ω scan	θ_max_ = 25.0°, θ_min_ = 2.1°
Absorption correction: multi-scan (*SADABS*; Bruker, 2004)	*h* = −13→11
*T*_min_ = 0.761, *T*_max_ = 0.846	*k* = −23→22
10244 measured reflections	*l* = −10→7

### Refinement

**Table:**

Refinement on *F*^2^	Secondary atom site location: difference Fourier map
Least-squares matrix: full	Hydrogen site location: inferred from neighbouring sites
*R*[*F*^2^ > 2σ(*F*^2^)] = 0.046	H-atom parameters constrained
*wR*(*F*^2^) = 0.120	*w* = 1/[σ^2^(*F*_o_^2^) + (0.0567*P*)^2^ + 0.3483*P*] where *P* = (*F*_o_^2^ + 2*F*_c_^2^)/3
*S* = 1.08	(Δ/σ)_max_ < 0.001
3534 reflections	Δρ_max_ = 0.33 e Å^−^^3^
222 parameters	Δρ_min_ = −0.34 e Å^−^^3^
312 restraints	Absolute structure: Flack (1983), 1499 Friedel pairs
Primary atom site location: structure-invariant direct methods	Flack parameter: 0.00 (10)

### Special details

**Table:**

Geometry. All e.s.d.'s (except the e.s.d. in the dihedral angle between two l.s. planes) are estimated using the full covariance matrix. The cell e.s.d.'s are taken into account individually in the estimation of e.s.d.'s in distances, angles and torsion angles; correlations between e.s.d.'s in cell parameters are only used when they are defined by crystal symmetry. An approximate (isotropic) treatment of cell e.s.d.'s is used for estimating e.s.d.'s involving l.s. planes.
Refinement. Refinement of *F*^2^ against ALL reflections. The weighted *R*-factor *wR* and goodness of fit *S* are based on *F*^2^, conventional *R*-factors *R* are based on *F*, with *F* set to zero for negative *F*^2^. The threshold expression of *F*^2^ > σ(*F*^2^) is used only for calculating *R*-factors(gt) *etc*. and is not relevant to the choice of reflections for refinement. *R*-factors based on *F*^2^ are statistically about twice as large as those based on *F*, and *R*- factors based on ALL data will be even larger.

### Fractional atomic coordinates and isotropic or equivalent isotropic displacement parameters (Å^2^)

**Table:**

	*x*	*y*	*z*	*U*_iso_*/*U*_eq_	
C1	0.4798 (4)	0.78198 (19)	0.8644 (4)	0.0536 (9)	
H1	0.5545	0.7992	0.8232	0.064*	
C2	0.4754 (4)	0.79762 (19)	1.0295 (4)	0.0619 (10)	
H2	0.3996	0.7797	1.0661	0.074*	
C3	0.5733 (5)	0.7571 (2)	1.1058 (6)	0.0907 (13)	
H3A	0.6499	0.7742	1.0735	0.109*	
H3B	0.5677	0.7645	1.2114	0.109*	
C4	0.5674 (6)	0.6817 (2)	1.0753 (6)	0.0968 (14)	
H4A	0.6345	0.6594	1.1219	0.116*	
H4B	0.4953	0.6633	1.1185	0.116*	
C5	0.5686 (6)	0.6664 (2)	0.9133 (6)	0.0897 (13)	
H5	0.6455	0.6807	0.8729	0.108*	
C6	0.4707 (4)	0.70649 (18)	0.8358 (4)	0.0651 (10)	
H6A	0.4756	0.6983	0.7305	0.078*	
H6B	0.3940	0.6903	0.8696	0.078*	
C7	0.5537 (6)	0.5895 (2)	0.8864 (7)	0.1183 (17)	
H7A	0.6122	0.5650	0.9423	0.177*	
H7B	0.5640	0.5799	0.7833	0.177*	
H7C	0.4756	0.5756	0.9167	0.177*	
C8	0.4770 (5)	0.8738 (2)	1.0660 (5)	0.0761 (12)	
H8	0.4168	0.8955	1.0038	0.091*	
C9	0.4415 (6)	0.8870 (3)	1.2264 (6)	0.1027 (15)	
H9A	0.3749	0.8587	1.2517	0.154*	
H9B	0.4200	0.9340	1.2381	0.154*	
H9C	0.5072	0.8765	1.2900	0.154*	
C10	0.5941 (6)	0.9084 (3)	1.0329 (7)	0.1110 (16)	
H10A	0.6546	0.8904	1.0967	0.166*	
H10B	0.5864	0.9565	1.0491	0.166*	
H10C	0.6156	0.9001	0.9321	0.166*	
C11	0.3749 (3)	0.81985 (15)	0.6456 (3)	0.0374 (7)	
H11	0.4534	0.8277	0.6018	0.045*	
C12	0.2880 (3)	0.87618 (15)	0.6067 (3)	0.0347 (7)	
C13	0.1874 (3)	0.84593 (15)	0.5583 (4)	0.0383 (7)	
C14	0.2086 (3)	0.77365 (15)	0.5426 (4)	0.0393 (7)	
C15	0.2405 (3)	0.99911 (15)	0.6191 (4)	0.0427 (8)	
H15	0.1863	0.9920	0.5359	0.051*	
C16	0.1683 (3)	1.00945 (19)	0.7577 (5)	0.0611 (10)	
H16A	0.1188	0.9704	0.7741	0.092*	
H16B	0.1193	1.0492	0.7469	0.092*	
H16C	0.2208	1.0154	0.8402	0.092*	
C17	0.3137 (3)	1.06179 (15)	0.5905 (4)	0.0428 (8)	
Cl1	0.04908 (8)	0.87823 (4)	0.51989 (12)	0.0612 (3)	
N1	0.3197 (2)	0.94048 (12)	0.6313 (3)	0.0440 (7)	
H1A	0.3922	0.9481	0.6563	0.053*	
O1	0.3799 (2)	0.81852 (11)	0.7981 (2)	0.0423 (5)	
O2	0.31972 (19)	0.75881 (10)	0.5881 (3)	0.0426 (6)	
O3	0.1433 (2)	0.72965 (11)	0.4945 (3)	0.0532 (6)	
O4	0.2496 (2)	1.10975 (12)	0.5305 (4)	0.0780 (10)	
H4	0.2919	1.1427	0.5122	0.117*	
O5	0.4163 (2)	1.06720 (11)	0.6217 (3)	0.0610 (7)	

### Atomic displacement parameters (Å^2^)

**Table:**

	*U*^11^	*U*^22^	*U*^33^	*U*^12^	*U*^13^	*U*^23^
C1	0.061 (2)	0.0474 (17)	0.0526 (19)	0.0069 (16)	−0.0111 (16)	0.0100 (15)
C2	0.080 (2)	0.0552 (19)	0.051 (2)	−0.0053 (18)	−0.0131 (18)	0.0075 (17)
C3	0.120 (3)	0.078 (2)	0.074 (2)	0.009 (2)	−0.036 (2)	0.007 (2)
C4	0.135 (3)	0.074 (3)	0.081 (3)	0.019 (3)	−0.034 (3)	0.021 (2)
C5	0.127 (3)	0.065 (2)	0.077 (3)	0.031 (2)	−0.025 (3)	0.016 (2)
C6	0.094 (3)	0.0458 (19)	0.056 (2)	0.0143 (19)	−0.0131 (19)	0.0062 (16)
C7	0.181 (4)	0.075 (3)	0.099 (3)	0.046 (3)	−0.034 (3)	0.014 (3)
C8	0.102 (3)	0.064 (2)	0.062 (2)	−0.001 (2)	−0.015 (2)	−0.005 (2)
C9	0.141 (4)	0.094 (3)	0.073 (3)	−0.006 (3)	−0.003 (3)	−0.020 (3)
C10	0.137 (4)	0.090 (3)	0.107 (3)	−0.031 (3)	−0.005 (3)	−0.008 (3)
C11	0.0426 (16)	0.0299 (15)	0.0397 (18)	−0.0006 (14)	−0.0029 (14)	0.0015 (13)
C12	0.0369 (15)	0.0272 (14)	0.0401 (17)	−0.0002 (13)	0.0019 (13)	0.0026 (13)
C13	0.0395 (16)	0.0291 (14)	0.0463 (19)	0.0002 (13)	−0.0049 (14)	−0.0001 (14)
C14	0.0437 (18)	0.0324 (16)	0.0418 (19)	−0.0012 (14)	−0.0017 (14)	−0.0025 (14)
C15	0.0397 (16)	0.0267 (15)	0.061 (2)	0.0010 (13)	−0.0075 (15)	0.0044 (15)
C16	0.053 (2)	0.048 (2)	0.083 (3)	−0.0019 (17)	0.0107 (18)	0.0009 (19)
C17	0.0399 (19)	0.0279 (15)	0.061 (2)	0.0008 (14)	−0.0058 (16)	0.0032 (15)
Cl1	0.0444 (5)	0.0433 (5)	0.0960 (8)	0.0070 (4)	−0.0225 (5)	−0.0125 (5)
N1	0.0358 (15)	0.0283 (14)	0.068 (2)	−0.0006 (12)	−0.0108 (14)	0.0035 (13)
O1	0.0457 (13)	0.0385 (12)	0.0426 (14)	0.0057 (10)	−0.0046 (10)	0.0046 (10)
O2	0.0428 (13)	0.0264 (11)	0.0585 (15)	0.0032 (10)	−0.0074 (10)	−0.0051 (10)
O3	0.0541 (14)	0.0321 (12)	0.0734 (17)	−0.0052 (11)	−0.0091 (13)	−0.0130 (12)
O4	0.0509 (15)	0.0357 (14)	0.147 (3)	−0.0056 (12)	−0.0219 (17)	0.0330 (18)
O5	0.0410 (15)	0.0330 (12)	0.109 (2)	−0.0023 (10)	−0.0109 (14)	0.0093 (14)

### Geometric parameters (Å, °)

**Table:**

C1—O1	1.462 (4)	C9—H9C	0.9600
C1—C6	1.509 (5)	C10—H10A	0.9600
C1—C2	1.529 (6)	C10—H10B	0.9600
C1—H1	0.9800	C10—H10C	0.9600
C2—C3	1.524 (6)	C11—O1	1.384 (4)
C2—C8	1.532 (6)	C11—O2	1.447 (4)
C2—H2	0.9800	C11—C12	1.518 (4)
C3—C4	1.509 (6)	C11—H11	0.9800
C3—H3A	0.9700	C12—N1	1.331 (4)
C3—H3B	0.9700	C12—C13	1.351 (4)
C4—C5	1.499 (8)	C13—C14	1.447 (4)
C4—H4A	0.9700	C13—Cl1	1.716 (3)
C4—H4B	0.9700	C14—O3	1.215 (4)
C5—C6	1.525 (6)	C14—O2	1.348 (4)
C5—C7	1.540 (7)	C15—N1	1.460 (4)
C5—H5	0.9800	C15—C17	1.504 (4)
C6—H6A	0.9700	C15—C16	1.510 (5)
C6—H6B	0.9700	C15—H15	0.9800
C7—H7A	0.9600	C16—H16A	0.9600
C7—H7B	0.9600	C16—H16B	0.9600
C7—H7C	0.9600	C16—H16C	0.9600
C8—C10	1.512 (7)	C17—O5	1.193 (4)
C8—C9	1.530 (7)	C17—O4	1.305 (4)
C8—H8	0.9800	N1—H1A	0.8600
C9—H9A	0.9600	O4—H4	0.8200
C9—H9B	0.9600		
			
O1—C1—C6	111.1 (3)	C8—C9—H9A	109.5
O1—C1—C2	106.2 (3)	C8—C9—H9B	109.5
C6—C1—C2	111.3 (3)	H9A—C9—H9B	109.5
O1—C1—H1	109.4	C8—C9—H9C	109.5
C6—C1—H1	109.4	H9A—C9—H9C	109.5
C2—C1—H1	109.4	H9B—C9—H9C	109.5
C3—C2—C1	108.5 (4)	C8—C10—H10A	109.5
C3—C2—C8	113.8 (4)	C8—C10—H10B	109.5
C1—C2—C8	114.0 (3)	H10A—C10—H10B	109.5
C3—C2—H2	106.7	C8—C10—H10C	109.5
C1—C2—H2	106.7	H10A—C10—H10C	109.5
C8—C2—H2	106.7	H10B—C10—H10C	109.5
C4—C3—C2	113.4 (4)	O1—C11—O2	111.2 (2)
C4—C3—H3A	108.9	O1—C11—C12	105.8 (2)
C2—C3—H3A	108.9	O2—C11—C12	104.1 (2)
C4—C3—H3B	108.9	O1—C11—H11	111.8
C2—C3—H3B	108.9	O2—C11—H11	111.8
H3A—C3—H3B	107.7	C12—C11—H11	111.8
C5—C4—C3	112.1 (4)	N1—C12—C13	134.1 (3)
C5—C4—H4A	109.2	N1—C12—C11	118.7 (3)
C3—C4—H4A	109.2	C13—C12—C11	107.1 (3)
C5—C4—H4B	109.2	C12—C13—C14	109.0 (3)
C3—C4—H4B	109.2	C12—C13—Cl1	131.5 (2)
H4A—C4—H4B	107.9	C14—C13—Cl1	119.5 (2)
C4—C5—C6	109.9 (4)	O3—C14—O2	121.1 (3)
C4—C5—C7	110.5 (4)	O3—C14—C13	129.3 (3)
C6—C5—C7	110.8 (5)	O2—C14—C13	109.6 (3)
C4—C5—H5	108.5	N1—C15—C17	109.0 (2)
C6—C5—H5	108.5	N1—C15—C16	111.8 (3)
C7—C5—H5	108.5	C17—C15—C16	109.1 (3)
C1—C6—C5	112.3 (4)	N1—C15—H15	109.0
C1—C6—H6A	109.1	C17—C15—H15	109.0
C5—C6—H6A	109.1	C16—C15—H15	109.0
C1—C6—H6B	109.1	C15—C16—H16A	109.5
C5—C6—H6B	109.1	C15—C16—H16B	109.5
H6A—C6—H6B	107.9	H16A—C16—H16B	109.5
C5—C7—H7A	109.5	C15—C16—H16C	109.5
C5—C7—H7B	109.5	H16A—C16—H16C	109.5
H7A—C7—H7B	109.5	H16B—C16—H16C	109.5
C5—C7—H7C	109.5	O5—C17—O4	124.7 (3)
H7A—C7—H7C	109.5	O5—C17—C15	124.2 (3)
H7B—C7—H7C	109.5	O4—C17—C15	111.1 (3)
C10—C8—C9	109.9 (4)	C12—N1—C15	124.9 (3)
C10—C8—C2	114.0 (4)	C12—N1—H1A	117.5
C9—C8—C2	111.6 (4)	C15—N1—H1A	117.5
C10—C8—H8	107.0	C11—O1—C1	116.8 (3)
C9—C8—H8	107.0	C14—O2—C11	109.2 (2)
C2—C8—H8	107.0	C17—O4—H4	109.5
			
O1—C1—C2—C3	176.5 (3)	N1—C12—C13—Cl1	−5.9 (6)
C6—C1—C2—C3	55.4 (5)	C11—C12—C13—Cl1	170.3 (3)
O1—C1—C2—C8	−55.6 (5)	C12—C13—C14—O3	−175.4 (3)
C6—C1—C2—C8	−176.7 (4)	Cl1—C13—C14—O3	5.8 (5)
C1—C2—C3—C4	−54.8 (6)	C12—C13—C14—O2	3.1 (4)
C8—C2—C3—C4	177.1 (4)	Cl1—C13—C14—O2	−175.7 (2)
C2—C3—C4—C5	55.4 (7)	N1—C15—C17—O5	−23.0 (5)
C3—C4—C5—C6	−53.4 (7)	C16—C15—C17—O5	99.4 (4)
C3—C4—C5—C7	−176.0 (5)	N1—C15—C17—O4	158.5 (3)
O1—C1—C6—C5	−175.7 (4)	C16—C15—C17—O4	−79.2 (4)
C2—C1—C6—C5	−57.5 (5)	C13—C12—N1—C15	4.8 (6)
C4—C5—C6—C1	55.4 (6)	C11—C12—N1—C15	−171.0 (3)
C7—C5—C6—C1	177.8 (5)	C17—C15—N1—C12	−156.0 (3)
C3—C2—C8—C10	56.0 (6)	C16—C15—N1—C12	83.2 (4)
C1—C2—C8—C10	−69.1 (5)	O2—C11—O1—C1	83.7 (3)
C3—C2—C8—C9	−69.2 (6)	C12—C11—O1—C1	−163.9 (3)
C1—C2—C8—C9	165.7 (4)	C6—C1—O1—C11	−67.5 (4)
O1—C11—C12—N1	69.8 (3)	C2—C1—O1—C11	171.3 (3)
O2—C11—C12—N1	−172.9 (3)	O3—C14—O2—C11	−177.5 (3)
O1—C11—C12—C13	−107.0 (3)	C13—C14—O2—C11	3.8 (3)
O2—C11—C12—C13	10.2 (3)	O1—C11—O2—C14	105.0 (3)
N1—C12—C13—C14	175.6 (3)	C12—C11—O2—C14	−8.4 (3)
C11—C12—C13—C14	−8.2 (4)		

### Hydrogen-bond geometry (Å, °)

**Table:**

*D*—H···*A*	*D*—H	H···*A*	*D*···*A*	*D*—H···*A*
N1—H1A···O5^i^	0.86	2.20	2.975 (4)	150
O4—H4···O3^ii^	0.82	1.86	2.655 (3)	164

Symmetry codes: (i) −*x*+1, −*y*+2, *z*; (ii) −*x*+1/2, *y*+1/2, −*z*+1.
